# Infantile Kawasaki Disease Mimicking Polyarteritis Nodosa With Severe Ischemic Complications: A Case Report

**DOI:** 10.7759/cureus.105460

**Published:** 2026-03-18

**Authors:** Nadia Mebrouk, Bouchra Chkirate

**Affiliations:** 1 Pediatrics Department, Pediatrics Cardiology Unit, Children’s Hospital, Ibn Sina University Hospital Center, Faculty of Medicine and Pharmacy of Rabat, Mohammed V University, Rabat, MAR; 2 Pediatrics Department, Rheumatology and Pediatric Internal Medicine Unit, Children’s Hospital, Ibn Sina University Hospital Center, Faculty of Medicine and Pharmacy of Rabat, Mohammed V University, Rabat, MAR

**Keywords:** coronary artery aneurysms, digital ischemia, infantile kawasaki disease, polyarteritis nodosa, systemic vasculitis

## Abstract

Kawasaki disease (KD) is an acute medium-vessel vasculitis that primarily affects young children. In infants, the clinical presentation is frequently atypical or incomplete, with signs appearing progressively and non-simultaneously, which may lead to delayed diagnosis and increase the risk of severe vascular complications. Rarely, KD may present with angiographic findings mimicking polyarteritis nodosa (PAN), further complicating the diagnostic assessment. We report the case of a four-month-old male infant admitted for prolonged fever associated with initially incomplete mucocutaneous manifestations and rapidly progressive digital ischemia. The staggered appearance of symptoms resulted in initial management for presumed infection and subsequent diagnostic delay. Imaging studies revealed multiple extracoronary aneurysms involving medium-sized arteries, distal arterial occlusions, and giant coronary artery aneurysms, suggesting a systemic vasculitis resembling PAN. However, upon careful reassessment of the clinical evolution, the patient's young age and the presence of coronary artery involvement supported the diagnosis of a severe form of infantile KD. Treatment with intravenous immunoglobulins, corticosteroids, and antithrombotic therapy led to clinical and biological improvement, with regression of vascular lesions on follow-up imaging. This case highlights the importance of early consideration of KD in febrile infants presenting with systemic vascular involvement to prevent irreversible ischemic complications.

## Introduction

Kawasaki disease (KD) is an acute, self-limited vasculitis of medium-sized arteries and remains the leading cause of acquired heart disease in children in developed countries [1,2]. The diagnosis of KD is primarily clinical, based on prolonged fever and characteristic mucocutaneous findings, including bilateral non-purulent conjunctivitis, polymorphous rash, oral mucosal changes, extremity changes, and cervical lymphadenopathy, as no specific diagnostic test is currently available [1,3]. In infants younger than six months, however, KD often presents in an incomplete or atypical manner, with nonspecific symptoms that may appear sequentially [4,5]. These atypical presentations frequently lead to diagnostic delay and inappropriate initial management, placing this age group at increased risk of severe cardiovascular and systemic vascular complications [5].

In severe cases, KD may be associated with widespread extracoronary arterial involvement, including aneurysms of medium-sized systemic arteries, such as the axillary, iliac, or renal arteries [6]. Such presentations can closely mimic polyarteritis nodosa (PAN), a necrotizing vasculitis of medium-sized arteries that may involve multiple organs but typically lacks the characteristic mucocutaneous features and coronary artery involvement seen in KD [2]. However, unlike PAN, KD typically affects young children and is characterized by prominent mucocutaneous manifestations and a marked predilection for coronary artery involvement. In contrast, PAN is a necrotizing vasculitis that more commonly affects older children or adults and is usually not associated with the typical mucocutaneous features seen in KD. This PAN-like presentation represents a significant diagnostic challenge, particularly in infants, in whom classic mucocutaneous features of KD may be absent or subtle [7]. Misclassification of KD as PAN can result in delayed initiation of disease-specific therapy and worse vascular outcomes [2].

Early differentiation between KD and PAN is crucial, as management strategies and outcomes differ substantially [2]. Timely administration of intravenous immunoglobulin (IVIG) in KD has been shown to significantly reduce the risk of coronary artery aneurysms and other vascular complications [8]. According to the American Heart Association (AHA), delayed diagnosis and treatment of KD are strongly associated with adverse cardiovascular outcomes, especially in infants and in patients with incomplete clinical presentations [9].

We report a severe case of infantile KD that initially presented as a systemic PAN-like vasculitis, leading to delayed IVIG therapy and the development of peripheral ischemic complications, including digital necrosis. This case emphasizes the importance of considering KD in the differential diagnosis of systemic vasculitis in infants and highlights the need for early recognition of PAN-like presentations of KD to ensure prompt treatment and prevent irreversible vascular damage.

## Case presentation

A four-month-old male infant with no significant past medical history was admitted to our tertiary care center for persistent high fever (39-40 °C) lasting for 23 days. The initial course of the illness was characterized by the progressive and non-simultaneous appearance of a generalized rash, bilateral non-purulent conjunctivitis, cheilitis, edema of the extremities, and perineal desquamation. Because these manifestations did not occur simultaneously, the diagnostic criteria for KD were not initially fulfilled, which led to several medical consultations and the initiation of empirical treatment for a presumed infection, without clinical improvement.

**Figure 1:**
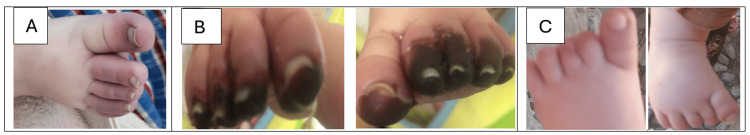
(A) Toe cyanosis at day 12 of fever onset. (B) Progression to distal ischemia and necrosis. (C) Clinical outcome after auto-amputation and healing

On the 12th day after the onset of fever, the infant developed cyanosis of the toes (Figure 1A), which progressively evolved over the following days into distal ischemia and subsequently bilateral digital necrosis (Figure 1B).

Due to the progressive worsening of the ischemic lesions, the infant was referred to our tertiary care center, where he was admitted on the 23rd day of illness.

At admission, the infant was febrile and irritable, with persistent cheilitis and severe distal ischemic lesions involving the toes. Retrospective analysis of the clinical course and detailed history revealed a complete mucocutaneous presentation compatible with KD.

Laboratory investigations revealed a severe inflammatory syndrome characterized by marked leukocytosis, extreme thrombocytosis, anemia, hypoalbuminemia, hyponatremia, and elevated liver enzymes, while an extensive infectious work-up remained negative (Table 1).

**Table 1:** Summary of laboratory results EBV: Epstein-Barr virus; CMV: cytomegalovirus; HBV: hepatitis B virus; HAV: hepatitis A virus; HCV: hepatitis C virus; HSV: herpes simplex virus; VZV: varicella-zoster virus

Category	Laboratory parameter	Patient value	Unit	Reference Range
Hematology	White blood cell count	30.98	×10⁹/L	6-17
	Platelet count	1091	×10⁹/L	150-450
	Hemoglobin	9.3	g/dL	9.5-13.0
	Mean corpuscular volume (MCV)	68	fL	74-108
	Mean corpuscular hemoglobin (MCH)	33	pg	25-35
Biochemistry	Serum albumin	30	g/L	35-50
	Serum sodium	130	mmol/L	135-145
Inflammatory markers	C-reactive protein (CRP)	95	mg/L	<5
	Erythrocyte sedimentation rate (ESR)	62	mm/h	0-10
	Fibrinogen	6.2	g/L	1.5-4.0
Liver enzymes	Alanine aminotransferase (ALT)	215	U/L	5-45
	Aspartate aminotransferase (AST)	124	U/L	5-45
Infection workup	Blood cultures	Negative	-	-
	Urine culture (ECBU)	Leukocyturia positive (10), without bacteriuria	-	-
	Viral serologies (EBV, CMV, HBV, HAV, HCV, HSV, VZV)	Negative	-	-
	Lumbar puncture	Negative	-	-

Autoimmune investigations, including antineutrophil cytoplasmic antibodies (ANCA) and complement levels, were within normal limits. Furthermore, the patient did not meet the diagnostic criteria for PAN, supporting the diagnosis of KD. Marked thrombocytosis is a common laboratory feature of KD, typically occurring during the subacute phase and reflecting intense inflammatory activity. Hyponatremia has also been associated with more severe inflammatory responses and may correlate with an increased risk of coronary artery involvement.

**Figure 2:**
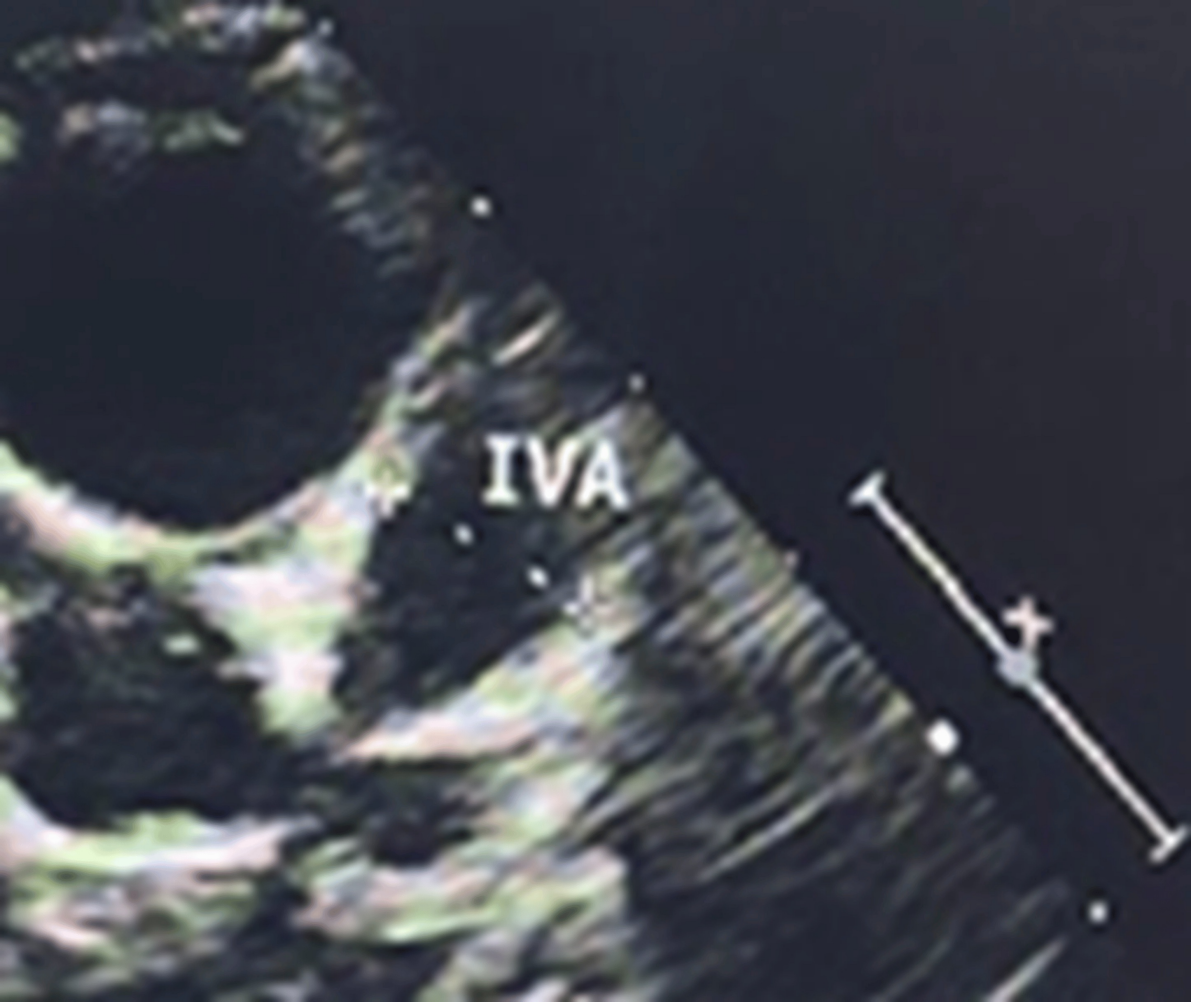
Transthoracic echocardiography revealing aneurysms of the left anterior IVA: isovolumic acceleration

Transthoracic echocardiography demonstrated multiple coronary artery aneurysms, including a giant aneurysm of the left anterior descending coronary artery measuring 8 mm and an aneurysm of the left circumflex coronary artery measuring 4 mm (Figure 2).

**Figure 3:**
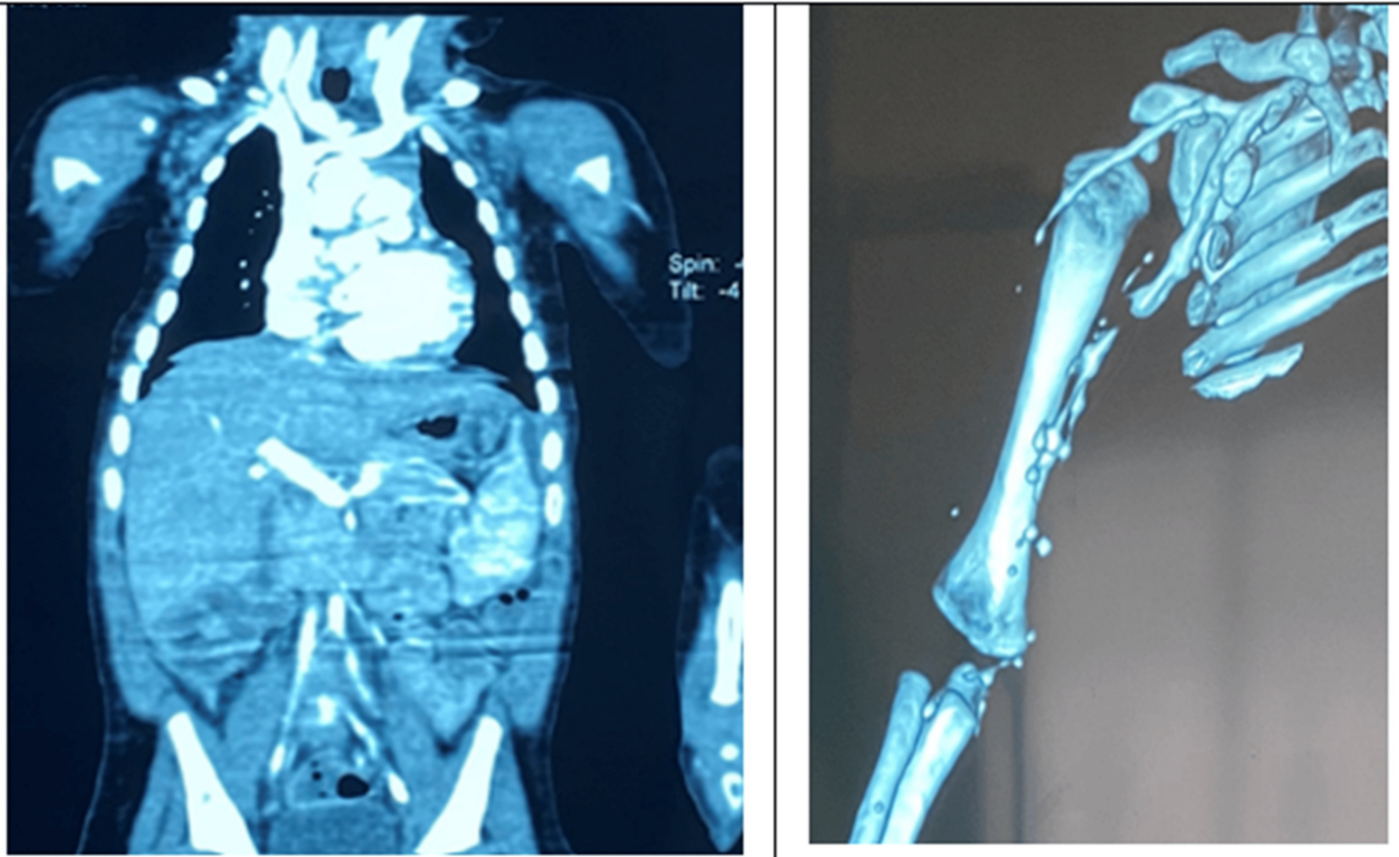
Computed tomography angiography demonstrating diffuse fusiform, saccular, and moniliform aneurysmal dilatations involving multiple medium-sized arteries

Vascular Doppler ultrasonography showed absent flow in the distal tibial arteries. Computed tomography angiography demonstrating diffuse fusiform, saccular, and moniliform aneurysmal dilatations involving multiple medium-sized arteries, consistent with a PAN-like systemic vasculitis (Figure 3). Skin biopsy demonstrated late fibrotic changes without evidence of active vasculitis.

IVIG was administered on the day of admission (day 23 of illness) at a dose of 2 g/kg as a single infusion, once the clinical findings strongly suggested KD. Although treatment was initiated beyond the optimal therapeutic window, it was considered necessary given the persistence of fever, marked irritability, and the presence of ongoing progressive vascular lesions. The patient was also treated with high-dose intravenous methylprednisolone pulses (30 mg/kg/day for three days), followed by oral corticosteroid therapy, in addition to antiplatelet therapy and anticoagulation with low-molecular-weight heparin. The clinical response was remarkable, with rapid resolution of fever, disappearance of the main clinical signs, and normalization of inflammatory markers. Follow-up imaging demonstrated a favorable evolution with partial regression of both coronary and extracoronary aneurysmal lesions. Despite distal auto-amputation of the necrotic toes, the overall clinical and vascular outcome was favorable (Figure 1C).

## Discussion

KD in young infants represents a well-recognized diagnostic challenge because clinical manifestations are frequently incomplete and atypical and appear sequentially rather than simultaneously [1,3,7]. Several studies have shown that infants younger than six months are less likely to fulfill classical diagnostic criteria at initial presentation and are therefore at increased risk of delayed diagnosis and inappropriate initial management [3,4,7]. This delay has been consistently associated with a higher incidence of coronary artery aneurysms and systemic vascular complications compared with older children [3,8].

The present case illustrates these difficulties, as the staggered appearance of mucocutaneous signs initially led to repeated evaluations for presumed infection, a scenario frequently described in infantile KD [7]. Laboratory findings in our patient, including marked leukocytosis, extreme thrombocytosis, hypoalbuminemia, hyponatremia, and elevated inflammatory markers, are typical of severe KD and have been associated with increased risk of vascular complications and IVIG resistance [1,3,9]. However, in the absence of simultaneous clinical criteria, these biological features may be insufficient to prompt early diagnosis in routine practice.

A particularly striking feature of this case was the presence of extensive extracoronary arterial aneurysms with distal occlusions, creating a radiological picture highly suggestive of PAN. Although coronary artery involvement is a hallmark of KD, systemic arterial aneurysms affecting medium-sized arteries have been increasingly reported in severe forms of the disease, especially in infants [2,6]. Orr et al. highlighted that extracoronary systemic arterial aneurysms in KD remain underrecognized and poorly characterized, representing an important evidence gap that may contribute to diagnostic confusion with PAN [6]. Similar PAN-like presentations of KD with renal, mesenteric, or peripheral arterial involvement have been described, emphasizing that systemic vasculitis does not exclude KD, particularly in young infants [2]. Although the systemic arterial involvement observed in our patient initially suggested PAN, the presence of mucocutaneous features and coronary artery aneurysms strongly supported the diagnosis of KD.

Distinguishing KD from true PAN is essential, as therapeutic strategies and prognostic implications differ significantly (Table 2).

**Table 2:** Key differentiating features between Kawasaki disease and polyarteritis nodosa IVIG: intravenous immunoglobulin

Feature	Kawasaki disease	Polyarteritis nodosa
Typical age	Infants and young children	Older children and adults
Fever	Prominent and prolonged	Variable
Mucocutaneous signs	Typical	Usually absent
Coronary artery involvement	Characteristic	Rare
Systemic medium-artery aneurysms	Rare but possible	Common
Response to IVIG	Typical	Not characteristic

PAN is typically associated with hypertension, renal involvement, and infectious triggers, such as hepatitis B, features that were absent in our patient [2]. In contrast, the presence of giant coronary artery aneurysms, the acute febrile course, and the rapid clinical and biological response to KD-specific therapy strongly supported the diagnosis of severe infantile KD [2-4,9]. Recent AHA guidelines stress the importance of coronary artery imaging and age-adapted clinical interpretation when evaluating suspected systemic vasculitis in infants [9].

Digital ischemia and gangrene represent rare but devastating complications of KD. Since the first descriptions by Tomita et al., peripheral ischemic manifestations have been reported almost exclusively in severe cases with delayed diagnosis, intense systemic inflammation, thrombocytosis, and thrombotic phenomena within aneurysmal vessels [5]. The mechanisms proposed include inflammatory arteritis, endothelial injury, aneurysm-related thrombosis, and hypercoagulability, all of which were present in our patient [3,5]. These complications underscore the need for aggressive and multidisciplinary management, including immunomodulatory therapy and antithrombotic treatment.

Importantly, recent data confirm that early administration of IVIG within the first 7-10 days of fever onset significantly reduces the risk of coronary and systemic arterial complications [8]. In infants with incomplete presentations, delayed recognition may therefore have irreversible consequences, as illustrated by the digital necrosis observed in this case despite subsequent clinical improvement.

Overall, this case reinforces existing literature highlighting that KD should remain a key diagnostic consideration in febrile infants presenting with systemic vasculitis and PAN-like angiographic findings. Careful reconstruction of the chronological evolution of clinical signs, systematic coronary artery assessment, and awareness of extracoronary vascular involvement are critical to establishing the correct diagnosis and initiating timely therapy to prevent catastrophic ischemic and cardiac complications.

## Conclusions

This case underscores the diagnostic challenge of KD in young infants, in whom clinical manifestations are often incomplete, atypical, and sequential. Severe systemic arterial involvement with a PAN-like angiographic pattern may represent a major diagnostic pitfall and delay appropriate treatment. Careful analysis of the clinical timeline, patient age, and systematic assessment for coronary artery involvement are essential to differentiate severe infantile KD from true PAN. Early recognition and prompt initiation of KD-specific immunomodulatory and antithrombotic therapy are critical to reducing the risk of coronary artery aneurysms and irreversible ischemic complications.

This case highlights the importance of maintaining a high index of suspicion for KD in febrile infants presenting with systemic vasculitis. Systemic medium-sized artery aneurysms in infants should not automatically be diagnosed as PAN without careful evaluation of the coronary arteries.
